# Contributions from Patients

**Published:** 1920-03-27

**Authors:** 


### CONTRIBUTIONS FROM PATIENTS.

**Mr. J. W. Pennyman, Chairman, North Ormesby Hospital, Middlesbrough.**

As a general proposition, I think many patients might well contribute. In the case of the North Ormesby Hospital (Middlesbrough) few patients could be asked to contribute, because of our present system, which, from experience, I very strongly recommend as an alternative scheme where suitable. It is, I believe, the usual practice in the north-east of England. It is this: The workmen in the various works, large and small, in our district agree to give us a fixed contribution, in some cases 1d. a week, in some 2d., in some 1½d., and they authorise their employers to deduct the sum from their wages and pay it over to us in bulk. Last year, out of a total income of £13,000, these workmen's contributions amounted to £9,000. (I give round figures, from memory.) The result is that we are not in debt and have money in hand. The works get books of tickets of admission, and have five seats on the Council, which consists of about twenty members.

Most of our cases come from the works in the district, and as they have already contributed, as I describe, we could not possibly ask them for a further contribution of 10s. a week.

Surely a system which is capable of producing nearly three-quarters of the necessary income of a hospital is not one to be lightly rejected without trial.

**Mr. Walter Mason, Hon. Secretary, South-Eastern Hospital for Children, Sydenham.**

I have read with interest the letters in regard to the London Hospital proposals. I personally consider that a charge should be made for in-patients where possible, but to draw a hard-and-fast line of a contribution of 10s. weekly is not practicable, often the patient will be the father of a family and the breadwinner; where is the 10s. to come from? I also consider that in this neighbourhood such a scheme would alienate many of our subscribers. We receive patients by free letters or by letters at reduced fees, and most subscribers appear to appreciate receiving letters according to the amount of their subscriptions, even if they do not always use them.

We have recently opened a paying ward (four cots) at a charge of not less than £2 2s. weekly, parents making their own arrangements with a member of the medical staff. This has already been appreciated, and adds to the fund of the hospital.

The ideal plan would be if in every case the home of the patient could be visited and for a small Committee to assess the patient's payment according to their circumstances. This would necessitate all hospitals having a lady almoner.

**Mr. H. Edgell Brown, Secretary, The Passmore Edwards Hospital for Willesden.**

Respecting the present critical financial state of London hospitals, I would like to state a few facts as seen from my point of view: (1) Hospitals are a necessity. (2) In the past the greatest supporters in bulk (*i.e.*, the most generous supporters according to their means) were the middle and professional class. (3) The only class who have benefited by having increased wages or salaries to meet the increased cost of living are the manual labouring classes. The professional and brain worker remain as before the war. These people have a very hard struggle to make both ends meet, and cannot be expected to continue their subscriptions to charities in the same measure as heretofore. (4) This loss to charities, hospitals in particular, has to be faced. It is for the benefit of the nation's health generally that disease must be treated and provision made for its treatment, or the future generation must suffer. This being the case, hospitals cannot be given up or their work cease on account of any lack of free and voluntary help. (5) As the State does not seem prepared to take them over, self-preservation points out another course, *viz.*, the obtaining of funds wherever and whenever they may be obtained—from the patients themselves. If a patient lived at home, he would at least have to buy food—why not pay to the hospital the amount received as benefit from the National Health Insurance. If it must be so, it is far better that the landlord should have a week or two rent owing to him than the possible loss of life or the risk of becoming a chronic invalid on the part of the patient. If the health of the working-classes once becomes undermined through lack of treatment we should soon deteriorate as a nation and lose our place in the world of commerce. When one is faced with a crisis in our hospitals as exists at the present time; sentiment must go by the board and the whole outlook must be treated from a business point of view. (6) If the masses did not approve of any attitude the hospitals adopted they would then force the hands of their local Members of Parliament, and, through them, the Government, whose duty it would then be to help hospitals to keep open their doors. No doubt many of the smaller hospitals in the suburbs could be made self-supporting by having a graduated scale of payment according to a patient's means and the requirements of the case.

